# Interstitial lung disease following combined CDK4/6 inhibitor therapy and radiotherapy in advanced breast cancer: a case report

**DOI:** 10.3389/fmed.2025.1661867

**Published:** 2025-10-31

**Authors:** Lanlan Guo, Yalan Dai, Mei Xiao, Zhiping Li, Yinyan Mao, Zhiquan Zhu, Xiaolu Xu, Peijian Peng

**Affiliations:** ^1^Department of Breast Oncology, The Cancer Center of the Fifth Affiliated Hospital of Sun Yat-sen University, Zhuhai, Guangdong, China; ^2^Key Laboratory of Oncology in South China, Department of Radiation Oncology, Sun Yat-sen University Cancer Center, Guangzhou, China; ^3^Department of Radiation Oncology, Sun Yat-sen University Cancer Center, Guangzhou, China

**Keywords:** breast cancer, cyclin 4 and 6 dependent kinase inhibitors, radiotherapy, interstitial lung disease, abemaciclib

## Abstract

**Background:**

Cyclin 4 and 6 dependent kinase inhibitors (CDK4/6i) have recently been approved for postmenopausal women diagnosed with hormone receptor–positive and HER2-negative metastatic breast cancer in combination with endocrine therapy. Research on the interaction of CDK4/6i and radiotherapy are scarce, but we observed some unexpected and severe toxicity, such as interstitial lung disease (ILD).

**Cases:**

Through blocking the transition from the G1 phase to the S phase (DNA synthesis phase), CDK4/6i inhibit tumor cell proliferation. The most common adverse event is neutropenia. Gastrointestinal toxicity, fatigue, QT Interval prolongation, increased liver enzymes, venous thromboembolic events, and ILD. Although ILD is very unlikely to occur, if suspected (e.g., worsening cough, dyspnea), interrupt treatment immediately and to evaluate the patient. In this study, we reported two cases of ILD in patients treated with the combination of radiotherapy and CDK4/6i. We also detailed the management strategy for patients who developed ILD, along with the subsequent clinical course and outcomes. Ultimately, with prompt and effective management, both patients showed improvement.

**Conclusion:**

These cases suggest that CDK4/6i may potentiate radiotherapy-associated pulmonary toxicity, and clinicians should exercise caution with this combination.

## Introduction

Since their introduction in 2017, cyclin-dependent kinase 4/6 inhibitors (CDK4/6i) have become a cornerstone in treating ER+/HER2- advanced or metastatic breast cancer (1). As a class of targeted anticancer drugs, these agents exert their therapeutic effect by targeting key cell cycle regulatory proteins. Inhibiting kinase activity, thereby blocking cell cycle progression from G1 to S phase and preventing tumor cell proliferation In HR+ breast cancer cells, growth signals such as estrogen activate cyclin D, which binds to CDK4/6 to form a complex. This complex phosphorylates the retinoblastoma (Rb) protein, releasing its inhibition of E2F transcription factors, thereby driving cell cycle progression from G1 to S phase for proliferation. CDK4/6i (e.g., palbociclib, ribociclib) reversibly bind to the ATP-binding site of CDK4/6, blocking the kinase activity. This maintains Rb in its unphosphorylated state, sustaining inhibition of E2F, eventually inducing G1 phase arrest and preventing tumor cell proliferation (2, 3). Importantly, CDK4/6-mediated cell cycle regulation contributes to the development of endocrine therapy resistance (3, 4).

The incorporation of CDK4/6i into treatment regimens for HR+/HER2- advanced breast cancer has significantly improved clinical outcomes, demonstrating benefits in progression-free survival (PFS), objective response rate (ORR), overall survival (OS), and quality of life (5). Based on pivotal PALOMA, MONARCH, and MONALEESA trials (6–12) encompassing eight clinical studies, palbociclib, abemaciclib, and ribociclib received FDA and EMA approval for use with aromatase inhibitors or fulvestrant in this patient population. Neutropenia was the common dose-limiting toxicity (DLT) observed with palbociclib and ribociclib in phase I safety evaluation. Furthermore, ribociclib could result in QTc interval prolongation of another DLT (13–15). By comparison, the common DLTs observed with abemaciclib were diarrhea and fatigue (16). The incidence of interstitial lung disease (ILD) caused by CDK4/6i is extremely low, but it requires great caution. Most of previous reports of ILD were from CDK4/6i alone. Compared with control groups, these studies have indicated a higher incidence of ILD among patients treated with ribociclib (17, 18), palbociclib (19), and abemaciclib (20, 21). Nevertheless, the pathogenesis of these adverse events remains poorly understood.

However, data regarding interactions between CDK4/6i and radiotherapy remain limited. Current evidence suggests radiotherapy may potentiate known CDK4/6i toxicities, particularly hematological effects like neutropenia and leukopenia (22). Current protocols typically recommend interrupting palbociclib during palliative radiotherapy for bone metastases, with a treatment pause spanning from 1 day before to 1 week after radiation (9, 23).

Preclinical studies indicate CDK4/6i may enhance radiotherapy efficacy through multiple mechanisms. Palbociclib appears to inhibit double-stranded DNA repair (24), while abemaciclib functions as a multifunctional radiation modifier (25). Retrospective clinical analyses have not identified increased hematological, dermatological, neurological, or gastrointestinal toxicity with combination therapy compared to CDK4/6i alone (26–29). The largest case series to date (*n* = 85) evaluating palbociclib or ribociclib with radiotherapy found no increased need for dose reduction or discontinuation due to adverse events (30). Nevertheless, larger prospective studies are needed to fully characterize long-term toxicities and evaluate potential dose-response relationships.

ILD represents a spectrum of pulmonary disorders characterized by progressive dyspnea, dry cough, and potentially severe bilateral fibrosis (honeycomb lung), which may progress to respiratory failure and cor pulmonale. Notably, no prior studies have specifically investigated ILD associated with combined CDK4/6i and radiotherapy in breast cancer patients. This study examines two cases of ILD occurring in HR+/HER2- advanced breast cancer patients treated with this therapeutic combination.

## Cases

### Patient 1

A 60-year-old woman was initially diagnosed with left-sided HR+/HER2-negative breast cancer. She underwent breast-conserving surgery and axillary lymph node dissection, which revealed invasive lobular carcinoma with ductal carcinoma *in situ*. Of the 19 dissected lymph nodes, none showed micrometastasis. She received six cycles of chemotherapy with doxorubicin (adriamycin) and cyclophosphamide. Due to poor post-operative wound healing, a left mastectomy was subsequently performed. She then continued endocrine therapy with tamoxifen for 5 years until experiencing a recurrence involving the right iliac crest, accompanied by intermittent pain. Palliative radiotherapy (60 Gy in 25 fractions) was administered to the affected iliac region.

At this point, her treatment regimen was switched to palbociclib (125 mg orally, 3 weeks on/1 week off) and anastrozole (1 mg daily). However, after 1.5 years, she developed vertigo, and brain MRI revealed multiple metastatic lesions in both cerebral and cerebellar hemispheres. She underwent palliative whole-brain radiotherapy (60 Gy/20 fractions) and transitioned to abemaciclib, exemestane, and fulvestrant for targeted endocrine therapy.

**Figure 1:**
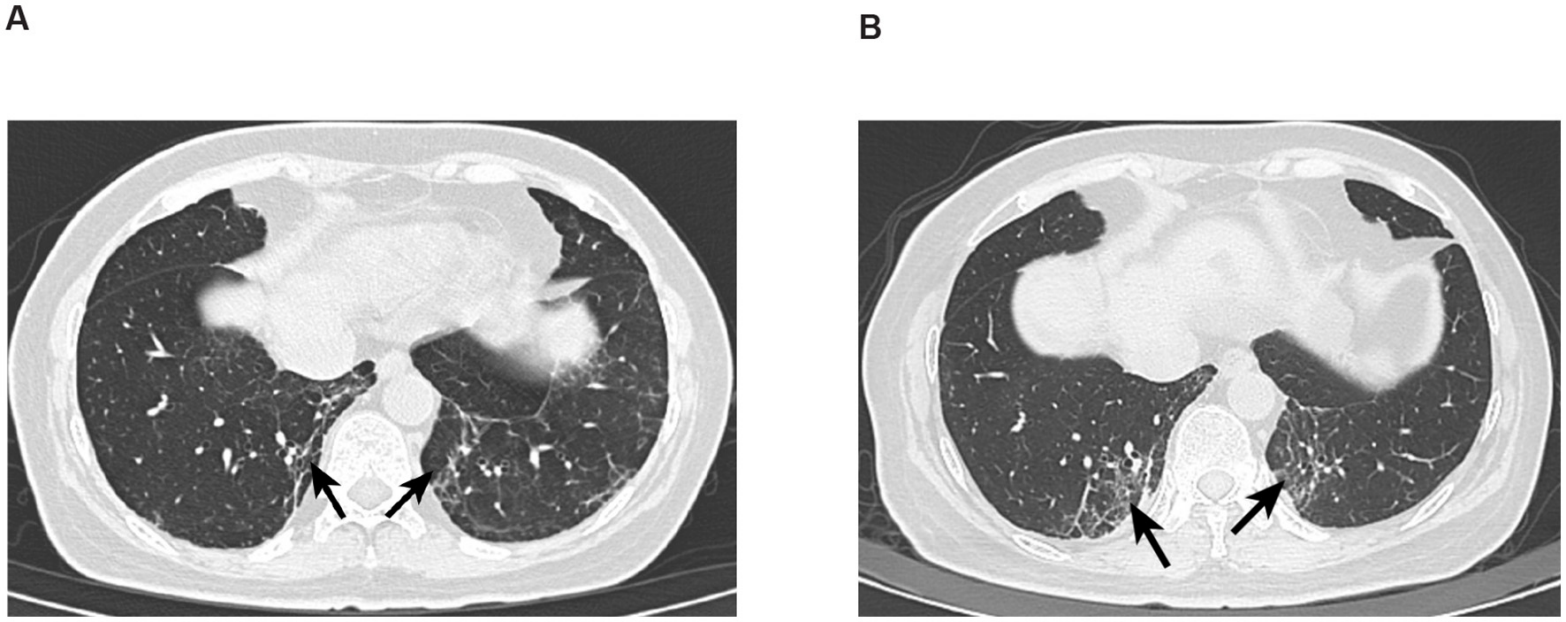
**(A)** CT image of Patient 1 with interstitial lung disease. Absent left breast, consistent with postoperative changes, generally unchanged from previous; follow-up and reexamination recommended. Scattered inflammatory organized foci in both lungs; interstitial inflammation in the lower lobes of both lungs has significantly increased compared to previous. **(B)** CT image of Patient 1 with interstitial lung disease after 4 months. Postoperative changes in the left breast, generally unchanged from previous, recommend follow-up and reexamination. Scattered inflammatory organized foci in both lungs, increased significantly from previous; improved reexpansion of the lower lobes of both lungs compared to prior, and the previously noted bilateral minimal pleural effusions have now resolved.

Five months later, she reported dizziness, localized swelling pain on the right parietal scalp, and intermittent nausea. Chest and abdominal CT showed worsening interstitial inflammation in both lower lung lobes (Figure 1A). Follow-up brain MRI indicated that while the cerebellar metastases had slightly regressed, the cerebral lesions had increased in both number and size. Given the progression, she received additional palliative brain radiotherapy (60 Gy/25 fractions) while continuing her existing drug regimen.

During radiotherapy, she experienced myelosuppression, urinary tract infection, and intestinal infection, prompting temporary suspension of abemaciclib and radiation therapy. Treatment included leukocyte/platelet-boosting therapy and antibiotics. Upon clinical improvement, radiotherapy and abemaciclib were resumed, though the latter was later replaced with dalpiciclib.

Two months later, repeat CT imaging demonstrated increased inflammatory and organizing focal lesions in both lungs (Figure 1B). Concurrently, she developed fever, rash, aggravated myelosuppression, and worsening dizziness. Following multidisciplinary evaluation, dalpiciclib was discontinued, and supportive care (anti-inflammatory agents, blood cell stimulants, and anti-dizziness medications) was initiated. The anti-inflammatory agents included levofloxacin 0.5 g QD (1 week) for anti-infection treatment, followed by oral prednisone (40 mg/d) for 2 months. Her condition gradually stabilized, allowing dalpiciclib to be reintroduced. She subsequently received three cycles of bevacizumab.

After the final dalpiciclib cycle, she presented to the emergency department with fever, productive cough, dyspnea, and confusion. CT scans revealed stable brain metastases but progressive pulmonary inflammation, including new consolidations in the right lower lobe and worsening organizing pneumonitis bilaterally. Despite aggressive symptomatic management, the patient succumbed to circulatory failure 1 month later.

### Patient 2

A 51-year-old premenopausal woman was diagnosed with ER-/PR+/HER2- invasive ductal breast cancer with lymph node metastases. She received four cycles of chemotherapy (liposomal paclitaxel + doxorubicin) followed by adjuvant radiotherapy. Seven years later, an elevated CA19-9 level and bone scintigraphy revealed abnormal radiotracer uptake in the sternum, suggestive of bone metastasis. She developed progressive sternal pain, which was managed with palliative radiotherapy and bone-modifying agents (e.g., zoledronate/denosumab). Seven months later, her chest pain worsened, accompanied by cough, productive sputum, chest tightness, and dyspnea. CT (chest/abdomen) demonstrated multiple pulmonary nodules (consistent with metastases) and widespread bone metastases (left parietal bone, sternal body, T3, T4, T10, T12 vertebrae, left iliac crest). After bone-protective therapy, she underwent three cycles of gemcitabine (1.6 g, d1/d8) + cisplatin (40 mg, d1–3) but subsequently declined further chemotherapy. She was switched to endocrine therapy with toremifene citrate alongside continued bone protection.

**Figure 2:**
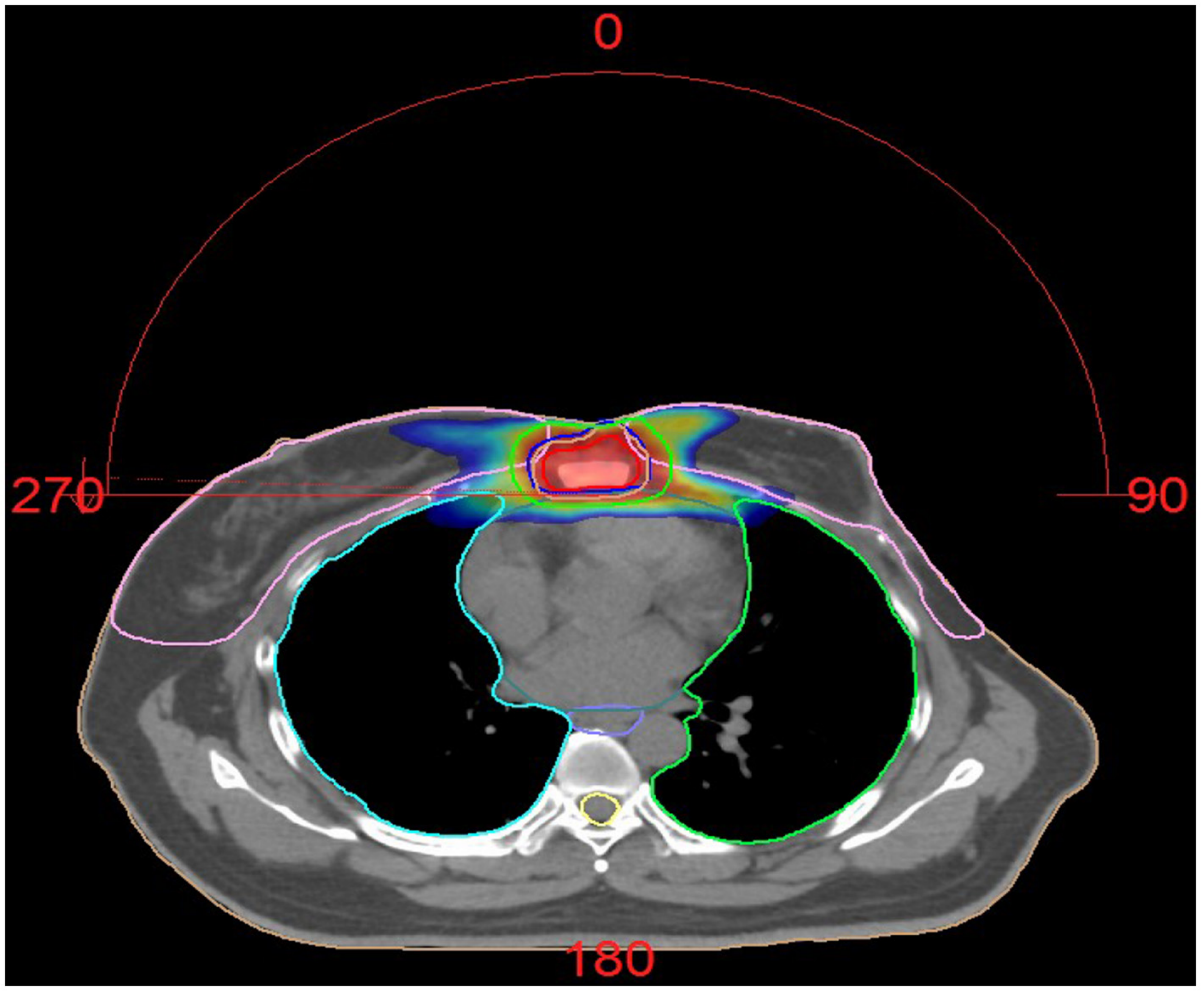
Dose distribution of Patient 2 treated with radiotherapy. Palliative radiotherapy was administered for the sternal metastasis using VMAT (Volumetric Modulated Arc Therapy) technique of IMRT (Intensity-Modulated Radiation Therapy). The plan was highly optimized to ensure adequate dose coverage to the target volumes while strictly limiting the radiation dose to surrounding normal organs (such as the heart, spinal cord, and lung tissue), keeping them within safe tolerance limits. The procedure was well tolerated. The prescribed dose was: GTV 60 Gy in 25 fractions, CTV 50 Gy in 25 fractions.

Five months later, she resumed menstruation, prompting the addition of goserelin (ovarian suppression therapy). However, due to intermittent goserelin non-adherence, menses recurred before therapy was reinstated. Three years later, she was hospitalized for cough and fever. CT showed stable subpleural lung nodules and right hilar lymphadenopathy, chronic inflammatory changes in bilateral upper/lower lobes, and unchanged sternal metastases. Influenza B screening was weakly positive (+/-). Antiviral therapy resolved her fever, but she continued to experience intermittent cough with white mucoid sputum. Six months later, repeat CT revealed worsening right lung inflammation and pleural thickening. Bronchoscopy identified a lesion in the right intermediate bronchus; biopsy and cytology confirmed metastatic breast cancer (supported by histology, IHC, and clinical history). She continued toremifene + goserelin. One year later, her regimen was adjusted to letrozole + goserelin. Six months thereafter, CT showed slight progression of lung nodules (right upper lobe, left lower lobe). She was switched to goserelin + anastrozole + palbociclib, though anastrozole was later replaced with exemestane due to gastric intolerance. For persistent sternal pain, she received palliative radiotherapy (60 Gy/25 fractions) (Figure 2).

**Figure 3:**
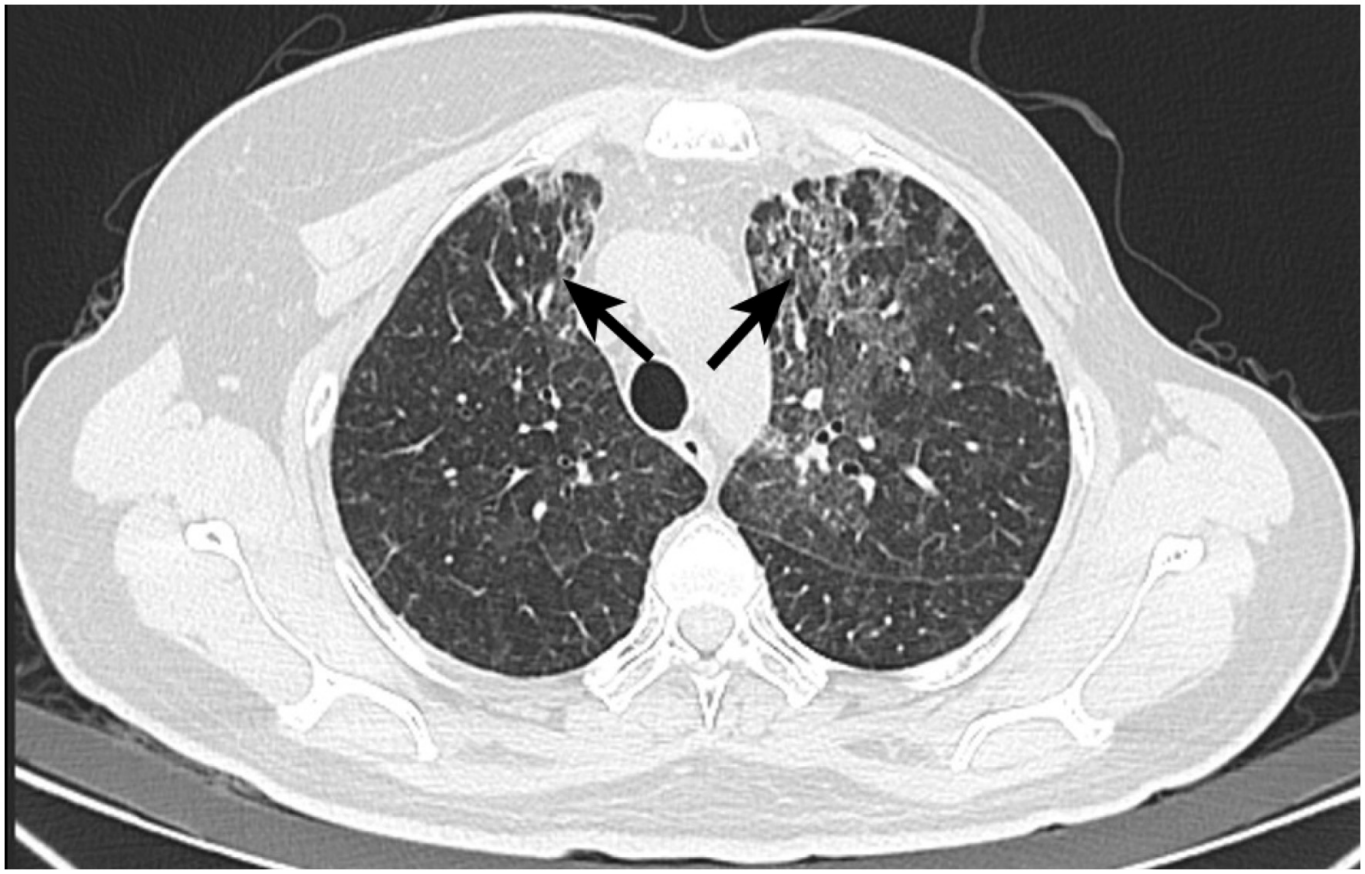
CT image of Patient 2 with interstitial lung disease. Postoperative changes after left breast cancer surgery, with streak and multiple high-density foci in the surgical area, generally unchanged in extent. Multiple faint small nodules and patchy opacities in both lungs, significantly increased from prior, metastasis cannot be excluded. Short-term follow-up after anti-inflammatory treatment is recommended. Possible interstitial lung disease is at the left lung apex. Scattered inflammatory organized foci in the right lung with corresponding pleural thickening, showing little change from previous.

**Figure 4:**
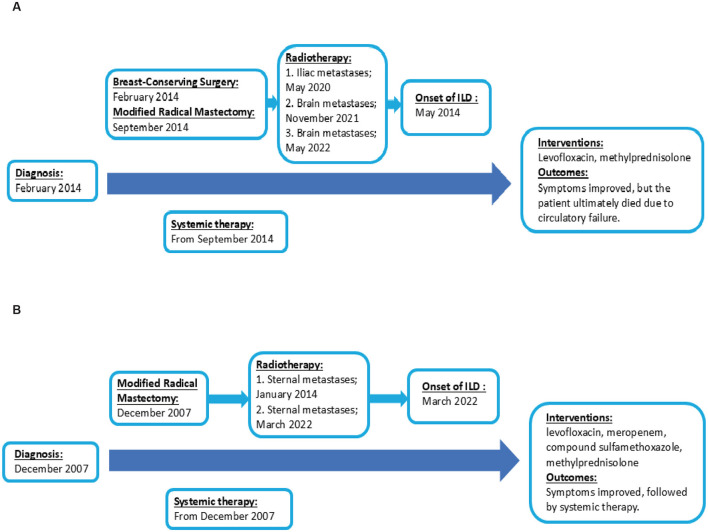
Timeline of diagnosis, interventions and outcomes. **(A)** The timeline of diagnosis, interventions and outcomes of Patient 1. **(B)** The timeline of diagnosis, interventions and outcomes of Patient 2.

Five months post-radiotherapy, she developed worsening cough, dyspnea, and frothy white sputum, requiring ICU admission. CT indicated new diffuse ground-glass opacities and interlobular septal thickening, suggestive of ILD (Figure 3). She received symptomatic management (antitussives/anti-inflammatories). The patient received levofloxacin 0.5 g QD (6 days), meropenem 0.5 g Q8H (8 days), and compound sulfamethoxazole tablets 0.96 g BID (9 days) for anti-infection treatment. Moreover, methylprednisolone (40 mg/d) was administered intravenously for 3 days, followed by oral prednisone (40 mg/d) for 1 month. Then, the patient had initiated goserelin + fulvestrant + dalpiciclib treatment, which she continues at present. Figure 4 showed the timeline of diagnosis, interventions and outcomes for two patients.

## Discussion

We present two cases of ILD in breast cancer patients receiving CDK4/6i alongside radiotherapy. The patient characteristics were shown in Table 1, with laboratory findings in Table 2. A prior study (31) described a 65-year-old metastatic breast cancer patient (liver and bone metastases) who developed fatigue and progressive dyspnea 3–5 months after initiating abemaciclib, culminating in acute hypoxic respiratory failure requiring hospitalization within 2–3 days. Among drug-induced ILD etiologies, antineoplastic agents are predominant. In breast cancer, targeted therapies such as trastuzumab deruxtecan, immune checkpoint inhibitors, everolimus, CDK4/6i, and PARP inhibitors are frequently implicated, while chemotherapeutics (e.g., bleomycin, platinum agents, methotrexate, taxanes, gemcitabine) are common culprits in broader oncology populations (32, 33).

**Table 1:** Patient characteristics.

**Characteristic**	**Patient 1**	**Patient 2**
Age	60	51
Gender	Female	Female
Relevant comorbidities	Type 2 diabetes	Type 2 diabetes
Radiation dose	GTV 60Gy/20F	GTV 60Gy/25F
		CTV 50Gy/25F
Target area	Brain metastases	Sternal metastases
Type of CDK4/6 inhibitors	Abemaciclib	Palbociclib
CDK4/6 inhibitors timing	Concurrent	Concurrent
Grade^#^ of ILD	Grade 2	Grade 2

^#^CTCAE Version 5.0

**Table 2:** Laboratory findings of 2 cases at ILD onset.

**Characteristic**	**Patient 1**	**Patient 2**
**Complete blood count**		
White blood cell counts (^*^10^9^/L)	7.62	9.38
Neutrophil counts (^*^10^9^/L)	4.1	5.46
Hemoglobin (g/L)	117	105
Platelet counts (/L)	125	228
C-reactive protein (mg/L)	16.93	58.66
Lactate Dehydrogenase (U/L)	238	277.3
**Liver function**		
Alanine aminotransferase (U/L)	32	36
Aspartate aminotransferase (U/L)	30	34
Gamma-glutamyl transferase (U/L)	40	184.5
Albumin (g/L)	39.5	48
**Kidney function**		
Creatinine (mg/dL)	39	64
Blood urea nitrogen (mmol/L)	5.7	6.3
Estimated glomerular filtration rate (mL/min/1.73 m^2^)	110	124
Uric acid (μmol/L)	136.5	366.3

In the Patient 1, the patient received palliative radiotherapy for brain metastases, followed by a switch from palbociclib to abemaciclib. Chest CT revealed worsening bilateral interstitial inflammatory lesions. In cancer patients, ILD remains a diagnosis of exclusion, particularly challenging during the COVID-19 pandemic. Differential diagnoses include sarcoidosis, infectious pneumonia, radiation pneumonitis, idiopathic pulmonary fibrosis, connective tissue disease-associated ILD, diffuse alveolar hemorrhage, and lymphangitic carcinomatosis. In this case, COVID-19/influenza PCR tests and bacterial/fungal cultures were negative. Despite symptomatic management (antitussives, mucolytics, anti-inflammatories), the patient succumbed to circulatory failure 6 months later. In the patient 2, during treatment with goserelin, exemestane, and palbociclib, the patient underwent palliative radiotherapy for sternal metastases. A multidisciplinary team (breast oncology, pulmonology, infectious diseases) excluded alternative causes, confirming ILD. Corticosteroids and supportive care in the ICU led to significant clinical improvement.

Therefore, it is evident that vigilant monitoring for ILD and other serious complications is imperative when combining CDK4/6 inhibitors with thoracic radiotherapy. Should ILD occur, prompt drug discontinuation, close surveillance of the patient's condition, and immediate therapeutic intervention are essential. An *in vivo* study indicated that palbociclib enhanced the recruitment of inflammatory cells—such as macrophages and T cells—into the bronchoalveolar lavage fluid, which may result from palbociclib-induced cell cycle arrest and associated cellular senescence (34, 35). Therefore, we hypothesize that radiotherapy provides the “first hit” by causing initial lung damage, while CDK4/6i deliver the “second hit” by crippling the lung's intrinsic repair capacity. The combination creates a perfect storm for progressive, unchecked inflammation and fibrosis, culminating in clinically significant ILD.

We hereby presented the first report of ILD and its prognosis in two breast cancer patients treated with a combination of CDK4/6i and radiotherapy. Patients tolerated CDK4/6i + radiotherapy without significant hematologic or intestinal toxicity. However, ILD manifested with debilitating respiratory symptoms (cough, dyspnea), severely impacting quality of life; one case required ICU admission. Notably, in the COVID-19 era, ruling out viral pneumonia is essential. The emergence of ILD introduced difficulties and challenges to the treatment of breast cancer patients, while our therapeutic experience also provided a valuable reference for subsequent research. More prospective clinical studies with larger sample sizes are needed for further validation. Additionally, extensive laboratory research is required to further explore the specific molecular mechanisms by which radiotherapy combined with CDK4/6is induces interstitial lung disease, thereby enabling early prevention and precise diagnosis and treatment. Our therapeutic experience also provides a valuable reference for subsequent research. However, the retrospective nature and reliance on medical records can lead to incomplete or inaccurate data. Furthermore, due to the fact that biopsies are not routinely performed in patients with ILD and radiation pneumonitis for further investigation, it is particularly challenging to conduct more in-depth research on these conditions.

## Conclusion

These cases highlight a potential interaction between CDK4/6 inhibitors and thoracic radiotherapy, resulting in interstitial lung disease. Clinicians should consider interrupting CDK4/6 therapy during radiotherapy and monitor patients closely for pulmonary toxicity.

## Data Availability

The raw data supporting the conclusions of this article will be made available by the authors, without undue reservation.
